# The compartmental restriction of cerebellar interneurons

**DOI:** 10.3389/fncir.2012.00123

**Published:** 2013-01-22

**Authors:** G. Giacomo Consalez, Richard Hawkes

**Affiliations:** ^1^Division of Neuroscience, San Raffaele Scientific InstituteMilan, Italy; ^2^Department of Cell Biology and Anatomy, Genes and Development Research Group, Faculty of Medicine, Hotchkiss Brain Institute, The University of CalgaryCalgary, AB, Canada

**Keywords:** Purkinje cell, stripe, zone, Golgi cell, basket cell, stellate cell, unipolar brush cell, granule cell

## Abstract

The Purkinje cells (PC's) of the cerebellar cortex are subdivided into multiple different molecular phenotypes that form an elaborate array of parasagittal stripes. This array serves as a scaffold around which afferent topography is organized. The ways in which cerebellar interneurons may be restricted by this scaffolding are less well-understood. This review begins with a brief survey of cerebellar topography. Next, it reviews the development of stripes in the cerebellum with a particular emphasis on the embryological origins of cerebellar interneurons. These data serve as a foundation to discuss the hypothesis that cerebellar compartment boundaries also restrict cerebellar interneurons, both excitatory [granule cells, unipolar brush cells (UBCs)] and inhibitory (e.g., Golgi cells, basket cells). Finally, it is proposed that the same PC scaffold that restricts afferent terminal fields to stripes may also act to organize cerebellar interneurons.

## Review of cerebellar compartmentation

The architecture of the adult cerebellar cortex is built around hundreds of modules (“stripes”), each comprising no more than a few hundred Purkinje cells (PC's: Hawkes et al., 1997; Apps and Hawkes, 2009: Figure 1). Along the rostrocaudal axis, the cerebellar cortex is divided into five transverse zones—the anterior zone (AZ: ~lobules I–V), central zone anterior (CZa: ~VI), central zone posterior (CZp: ~VII), posterior zone (PZ: ~VIII–IX), and nodular zone (NZ: ~X). Transverse zones and zonal boundaries are revealed by expression patterns (e.g., Odutola, 1970; Prasadarao et al., 1990; Eisenman and Hawkes, 1993; Millen et al., 1995; Alam et al., 1996; Ozol et al., 1999; Armstrong et al., 2000; Eisenman, 2000; Logan et al., 2002; Marzban et al., 2008; etc.), reflect patterns of cell death in many genetic mutations or toxic insults [reviewed in Sarna and Hawkes (2003)], and coincide with boundaries in the actions of mutations that disrupt cerebellar development and structure (Herrup and Wilczynsk, 1982; Hess and Wilson, 1991; Napieralski and Eisenman, 1993, 1996; Ackerman et al., 1997; Armstrong and Hawkes, 2001; Beirebach et al., 2001; etc.).

**Figure 1:**
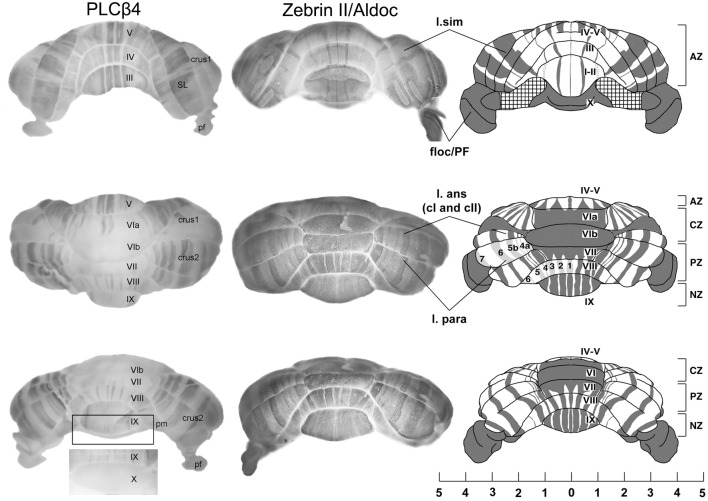
**An overview of cerebellar compartmentation.** Whole mount views of the complementary stripe arrays revealed by immunoperoxidase-staining for phospholipase cβ4 (PLCβ4) and zebrin II/aldolase C in the adult mouse. (Views: above—anterior; middle—dorsal; lower—posterior). Abbreviations: l. sim, lobulus simplex; floc/PFI, flocculus and paraflocculus; l. ans (c1 and c2), lobulus ansiformis, crus 1 and crus 2; l. para/pm, lobulus paramedianus; SL, lobulus simplex; AZ, anterior zone; CZ, central zone; PZ, posterior zone; NZ, nodular zone; lobules are indicated by Roman numerals (I–X). Scale bar is in mm [Adapted from Sarna et al. (2006) and Sillitoe and Hawkes (2002)].

Each transverse zone is further subdivided from medial to lateral into stripes. For example, Figure 1 shows alternating zones and stripes in cerebella immunostained for zebrin II (Brochu et al., 1990 = aldolase C (Aldoc)—Ahn et al., 1994; Hawkes and Herrup, 1996; Sillitoe and Hawkes, 2002) and phospholipase C (PLC)β4—Sarna et al., 2006). Many molecular markers co-localize with either the zebrin II+ or PLCβ4+ stripes [e.g., reviewed in Sillitoe et al. (2011); Sillitoe and Hawkes (2013)]. Furthermore, other markers reveal subdivisions within stripes (e.g., the patterns of afferent terminal fields: Akintunde and Eisenman, 1994; Ji and Hawkes, 1994, 1995; etc.) and additional PC subtypes within the zebrin II+/− families [e.g., heat shock protein (HSP)25: Armstrong et al. (2000)], the *L7/pcp2* transgene (Oberdick et al., 1993; Ozol et al., 1999) and human natural killer cell antigen 1 (HNK1: Eisenman and Hawkes, 1993; Marzban et al., 2004 identify subsets of zebrin II+ PCs.). The pattern of zones and stripes is symmetrical about the midline, highly reproducible between individuals and insensitive to experimental manipulation [see below, and reviewed in Larouche and Hawkes (2006); Apps and Hawkes (2009)]. The implication is that the adult cerebellar cortex of the mouse is highly reproducibly subdivided into several hundred distinct stripes with >10 distinct PC molecular phenotypes (Hawkes, 1997; Apps and Hawkes, 2009).

Transverse zones and parasagittal stripes are important because cerebellar patterning influences all aspects of cerebellar organization and function. The most-studied example is that the terminal fields of both climbing fibers and mossy fibers are aligned parasagittally with stripes of PCs (climbing fibers: Gravel et al., 1987; Voogd and Ruigrok, 2004; Sugihara and Quy, 2007; etc.; mossy fibers: Gravel and Hawkes, 1990; Akintunde and Eisenman, 1994; Ji and Hawkes, 1994; Sillitoe et al., 2003; Armstrong et al., 2009; Gebre et al., 2012; etc.).

The molecular topography of the cerebellar cortex correlates nicely with the functional maps [see Apps and Garwicz (2005); Apps and Hawkes (2009)]. For example, mossy fiber tactile receptive field boundaries correlate well with zebrin II+/− stripe boundaries [Chockkan and Hawkes, 1994; Hallem et al., 1999: see also Chen et al. (1996)]. More recently, Wylie et al. have demonstrated an elegant correlation between PC stripes and complex spike activity boundaries associated with optic flow in the pigeon vestibular zone (e.g., Graham and Wylie, 2012). The reproducible association of function with specific stripes also presents a potential substrate for function-specific adaptations at the molecular level. For instance, many of the molecules thought to mediate synaptic transmission and long-term depression at the parallel fiber-PC synapse show stripe restriction [including metabotropic glutamate receptors (Mateos et al., 2001), excitatory amino acid transporter 4 (Dehnes et al., 1998), PLC (Tanaka and Kondo, 1994; Sarna et al., 2006), protein kinase C (Chen and Hillman, 1993; Barmack et al., 2000), neuroplastin (Marzban et al., 2003), GABA receptors (Chung et al., 2008a), and so on]. Consistent with this hypothesis, electrophysiological studies have confirmed differences in parallel fiber-PC synaptic behavior between stripes (e.g., Wadiche and Jahr, 2005; Paukert et al., 2010; Ebner et al., 2012).

Thus, both patterns of gene expression and functional maps in the cerebellum seem to share a common architecture. The present review considers some of the evidence that PC stripe architecture also restricts the distributions of cerebellar interneurons. We begin with an overview of cerebellar pattern formation during development, then discuss the origins and development of the various cerebellar interneurons, review the evidence that interneurons are restricted to particular zones and stripes, and conclude by proposing the general hypothesis that interactions between interneurons and PCs during development are an important mechanism that restrict interneuron distributions.

Because we argue that much cerebellar patterning is built around a PC zone and stripe scaffold, we begin with a brief review of the origins of PC zones and stripes [reviewed in Herrup and Kuemerle (1997); Armstrong and Hawkes (2000); Larouche and Hawkes (2006); Sillitoe and Joyner (2007); Apps and Hawkes (2009); Dastjerdi et al. (2012); Sillitoe and Hawkes (2013)]. The cerebellar primordium arises from the rostral metencephalon between E8.5 and E9.5 (e.g., Wang et al., 2005; Sillitoe and Joyner, 2007: all timings are for mice). It houses two distinct germinal matrices—the dorsal rhombic lip (RL) and the ventral ventricular zone (VZ) of the 4th ventricle. Genetic fate mapping shows that a *Ptf1a* expressing domain in the VZ gives rise to all PCs (Hoshino et al., 2005; Hoshino, 2006). The *Ptf1a*+ VZ is not homogenous and gene expression differences further subdivide it (including *Ascl1, Neurogenin 1/2, Lhx1/5*, etc.—Chizhikov et al., 2006; Salsano et al., 2007; Zordan et al., 2008). PCs undergo terminal mitosis in the VZ between E10 and E13 (Miale and Sidman, 1961).

Adult PC zebrin II+/− phenotypes are specified early in development and birthdating studies in mice have identified two PC populations—an early born subset (E10–E11.5) mostly destined to become zebrin II+ and late-born subset (E11.5–E13) destined to become zebrin II—(Hashimoto and Mikoshiba, 2003; Larouche and Hawkes, 2006; Namba et al., 2011). Many experimental interventions—*in vitro* culture models, cerebellar transplants, afferent lesions, sensory deprivation, etc.—have been used to try to alter adult PC zebrin II+/− phenotypes, but these have always proved ineffective [reviewed in Larouche and Hawkes (2006)]. In fact, the only experimental manipulation known to alter PC subtype identity is deletion of the atypical helix-loop-helix transcription factor *Early B-cell Factor 2* (*Ebf2*), a repressor of the zebrin II+ phenotype (Croci et al., 2006; Chung et al., 2008b).

Postmitotic PCs migrate out of the VZ and stack in the cortical transitory zone with the earliest-born located dorsally and the youngest ventrally. Subsequently, the PCs reorganize to yield a stereotyped array of embryonic clusters with multiple molecular phenotypes [E14–E18: reviewed in e.g., Herrup and Kuemerle (1997)]. Starting at around E18, the embryonic clusters disperse, triggered by Reelin/Disabled-1 (Dab1) signaling (e.g., Armstrong and Hawkes, 2000; Larouche and Hawkes, 2006; Apps and Hawkes, 2009). As the clusters disperse into adult stripes the PCs spread to form a monolayer. Because dispersal occurs primarily in the anteroposterior plane, the clusters string out into long parasagittal stripes (e.g., Marzban et al., 2007).

The embryonic PC clusters are the targets for ingrowing climbing and mossy fiber afferents. Climbing fibers from the contralateral inferior olive enter the cerebellar cortex prenatally (Sotelo, 2004), and contact with PC clusters can be identified from birth (e.g., Mason et al., 1990). It appears that as the PC clusters disperse into parasagittal stripes the climbing fiber terminal fields ride along with them, thereby maintaining the embryonic topographical relationship and assuring a reproducible coupling between specific subnuclei of the inferior olivary complex and specific PC stripes [reviewed in Ruigrok (2011)]. Postnatally, extensive pruning of the climbing fiber projection occurs until each PC receives input from only one cell in the inferior olive, but this does not seem to contribute significantly to the refinement of the topography (Crépel, 1982). A similar sequence of events also patterns the mossy fiber projections, which are found in direct association with embryonic PC clusters from (*circa* E15: Grishkat and Eisenman, 1995; and possibly earlier—e.g., Morris et al., 1988). In the adult cerebellar cortex mossy fibers do not directly contact PCs. Rather, between P0 and P20, as the granular layer matures, mossy fiber afferents detach from the PCs and form new synapses with local granule cells. As a result, mossy fiber terminal fields retain their alignment with the overlying PC stripes (e.g., Gravel and Hawkes, 1990; Matsushita et al., 1991; Akintunde and Eisenman, 1994; Ji and Hawkes, 1994, 1995; Apps and Hawkes, 2009).

The aim of this review is to assess the evidence first that cerebellar interneurons show restriction and secondly to review the hypothesis that the PC architecture is the template around which they organize. This is a straightforward extension of the model previously espoused for the development of cerebellar afferent topography (e.g., Sotelo, 2004). The main classes of cerebellar interneurons are granule cells and unipolar brush cells (UBCs; glutamatergic—excitatory), and Golgi, stellate, and basket cells (GABAergic—inhibitory). In addition, there are several other types of inhibitory interneuron—Lugaro cells, Chandelier cells, etc. [see Schilling et al. (2008)]—but nothing is known of their patterns of restriction and they will not be considered further below.

## The embryological origins of cerebellar interneurons

**Figure 2:**
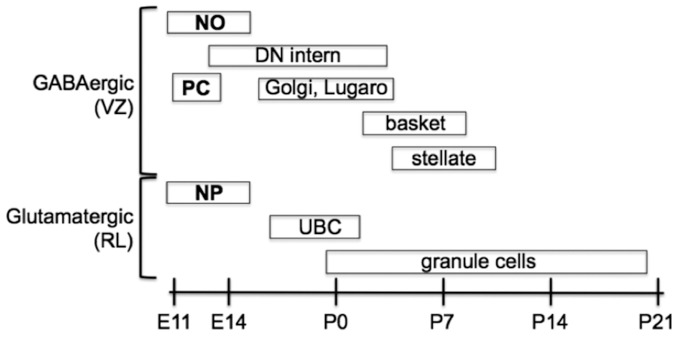
**Temporal sequence of birth for the main types of neurons that populate the adult cerebellum.** Projection neurons are in boldface, interneurons are in normal text. Projection neurons of the cerebellar nuclei and cortex are the first to be born at the outset of cerebellar neurogenesis. These include glutamatergic nuclear projection neurons (NP) derived from the rhombic lip (RL) and GABAergic nucleo-olivary projection neurons (NO) and Purkinje cells (PC) derived from the ventricular zone (VZ). Local interneurons (of both neurotransmitter phenotypes) are born during late embryonic and early postnatal development. GABAergic interneurons are generated according to an inside-out sequence, occupying the deep nuclei first, and then the granular and molecular layer [Modified from Carletti and Rossi (2008)].

Upon completion of early cerebellar patterning, neurogenesis begins. Two germinative compartments are established, the VZ and the rostral RL. In the mouse, this second phase of cerebellar development starts between E9 and E11 and proceeds for many days, giving rise to the different classes of cerebellar cells (Figure 2). At the onset of neurogenesis, the cerebellar primordium consists of two symmetric bulges extending dorsally and laterally from the midline of rhombomere one. These two halves are fated to eventually fuse at the midline, giving rise to a single dorsal formation spanning, and eventually exceeding, the width of the 4th ventricle. The inner and outer germinal layers of the cerebellar plate constitute the VZ and the RL, respectively (Altman and Bayer, 1997).

A series of studies conducted since 1990 have unveiled the origin of GABAergic and glutamatergic neurons that populate the cerebellar primordium and, eventually, the adult cerebellar cortex. The development of RL-derived progenitors is affected by signals produced by the roof plate (Alder et al., 1999; Millonig et al., 2000; Chizhikov et al., 2006). These progenitors soon become positive for the proneural gene *Atoh1/Math1* (Machold and Fishell, 2005; Wang et al., 2005). Targeted disruption of *Atoh1* virtually ablates the entire repertoire of cerebellar glutamatergic neurons (Jensen et al., 2004; Wang et al., 2005). Progenitor cells originating in the RL first migrate tangentially, dispersing over the dorsal surface of the cerebellar primordium, and then move radially into the cortex or cerebellar nuclei (CN). The first cells to migrate tangentially from the RL (*circa* E10.5) give rise to glutamatergic projection neurons of the CN (Machold and Fishell, 2005). They enter the primordium from just below its surface, giving rise to a transient structure sometimes referred to as nuclear transitory zone (NTZ), which will evolve into the CN. Shortly thereafter (*circa* E11–E14), a second echelon of RL-derived glutamatergic progenitors disperses by tangential migration to populate the external granular layer (EGL). These cells are fated to give rise to granule cell neurons only (Hallonet et al., 1990; Alvarez Otero et al., 1993). Starting shortly before birth, these progenitors undertake a long phase of clonal expansion in the EGL (between E17 and P20), under control of signals secreted by PCs (Smeyne et al., 1995; Dahmane and Ruiz-i-Altaba, 1999; Wallace, 1999; Wechsler-Reya and Scott, 1999; Lewis et al., 2004).

In addition to CN neurons and GCs, a third population of glutamatergic neurons originates in the embryonic cerebellum between E15 and E17: the so-called UBCs. UBCs are glutamatergic interneurons of the granular layer, with small somata, mossy fiber-like axon terminals, and brush-like dendrites (Altman and Bayer, 1977; Mugnaini and Floris, 1994; Diño et al., 1999, 2000, 2001; Nunzi and Mugnaini, 2000; Nunzi et al., 2001, 2002). Like granule cell progenitors, UBCs originate in the RL (Englund et al., 2006), and migrate inwards to invade the white matter of prospective lobule X. From there they disperse, populating the granular layer and extending glutamatergic axons akin to mossy fibers to form synapses with granule cell dendrites.

Unlike glutamatergic neurons, all GABAergic neurons of the cerebellum originate in a ventral germinative epithelium lining the 4th ventricle, called the VZ and recent evidence indicates that, as for granule cell proliferation, VZ progenitor proliferation is also controlled by *sonic hedgehog* (Huang et al., 2010). Projection neurons are generated first and local interneurons are born during late embryonic and early postnatal life (Miale and Sidman, 1961; Pierce, 1975; Altman and Bayer, 1997; Morales and Hatten, 2006). While GABAergic projection neurons only proliferate in the VZ, interneurons derive from progenitors that delaminate from the VZ into the prospective white matter. Projection neurons (PCs and nucleo-olivary neurons) proliferate in the VZ and become committed to their fate at early stages of development, acquiring their mature subtype phenotypes through cell-autonomous mechanisms [for example, see Florio et al. (2012)]. Conversely, interneurons derive from progenitors that delaminate into the prospective white matter, where they develop in an inside-out progression (CN to granular layer to molecular layer) from a single pool of progenitors. While sojourning in the white matter, their fate choices, production rates, and differentiation schedules remain flexible and are largely dependent on stage-specific extracellular cues (Leto et al., 2006, 2009).

In regard to gene expression, all VZ-derived progenitors express *Ptf1a*, a gene encoding a bHLH transcription factor, as shown by targeted inactivation studies (Hoshino et al., 2005; Pascual et al., 2007). *Ptf1a*+ progenitors start regulatory cascades leading to the expression of other proneural genes (Zordan et al., 2008; Dastjerdi et al., 2012; Consalez et al., 2013). Cerebellar *Ascl1/Mash1*+ precursors give rise to PCs and to all GABAergic interneurons (Kim et al., 2008; Grimaldi et al., 2009; Sudarov et al., 2011). Precursors expressing *neurogenin 1* (*Neurog1*) become PCs or cortical GABA interneurons (Kim et al., 2008; Lundell et al., 2009). *Neurog2*+ precursors give rise to PCs and GABAergic CN neurons, including presumptive nucleo-olivary neurons and interneurons (Florio et al., 2012). Two subsets originate from that population. The first, located anteriorly and medially, starts expressing the proneural gene *Neurog1* and gives rise to cortical interneurons of the GL (Golgi, Lugaro) and ML (basket, stellate) (Lundell et al., 2009; Kim et al., 2011). The second group, positive for *Neurog2*, gives rise to CN interneurons (Florio et al., 2012). While the cell surface marker Neph3 is expressed throughout the VZ, including interneuron progenitors, E-cadherin (Cdh1) is differentially expressed, with higher levels found on the surface of mitotic PC progenitors (Mizuhara et al., 2009). To date, it is not clear if some progenitors co-express *Neurog1* and *Neurog2*.

## Zone and stripe boundaries restrict cerebellar interneurons

### Granule cells

The most plentiful cerebellar interneuron is the granule cell, which comprises almost all the neurons of the cerebellum. Granule cells receive their input from mossy fibers (mostly directly but in some cases via UBCs), and synapse in the molecular layer as parallel fiber synapses on PC dendrites and inhibitory interneurons. The development of granule cells has been studied extensively [e.g., reviewed in Chédotal (2010); Butts et al. (2011); Hashimoto and Hibi (2012)]. After several proliferative cycles, granule cell precursors located in the outer part of the EGL exit the cell cycle, express differentiation markers, and modify their repertoire of adhesion molecules (e.g., Xenaki et al., 2011). Through these surface molecules, in particular astrotactin (Edmondson et al., 1988), they contact the distal processes of Bergmann glia and undertake a centripetal radial migration in the course of which they begin to populate the granular layer, migrating inwards across the PC layer while extending T-shaped axons (future parallel fibers) orthogonal to their migration path (Altman and Bayer, 1997). Upon their arrival in the granular layer, granule cells receive inputs from mossy fiber presynaptic terminals, and trans-synaptically induce their maturation by secreting *Wnt* family ligands (Hall et al., 2000).

#### Restriction

Several lines of evidence point to an elaborate parcellation of the granular layer, with evidence of restriction both into transverse zones and parasagittal stripes. Differences in gene expression have revealed multiple granule cell subtypes [e.g., *Otx1/2*—Frantz et al., 1994; nicotinamide adenine dinucleotide phosphate (NADPH)-oxidase—Hawkes and Turner, 1994; fibroblast growth factor (FGF)1—Alam et al., 1996; Eph receptors and Ephrins—Rogers et al., 1999, etc.; reviewed in Hawkes and Eisenman (1997); Ozol and Hawkes (1997)]. Multiple granule cell subtypes can be explained in two broad ways: either they represent granule cell lineages or they are a secondary response to their local environment (for example, the local mossy fibers or PCs). In many cases, we cannot distinguish these possibilities. However, the analysis of murine embryonic stem cell chimeras has revealed two consistent granule cell lineage boundaries, one located close to the AZ/CZ PC boundary and a second to the PZ/NZ boundary (Hawkes et al., 1999: Figure 3). An AZ/CZ granule cell boundary can also be seen in the granular layer of several mouse mutants (e.g., *scrambler*—Goldowitz et al., 1997; *disabled*—Gallagher et al., 1998), and studies with *weaver* (*wv/wv*) X +/+ and *M. musculus* X *M. caroli* chimeras also revealed developmental boundaries at these sites (Goldowitz, 1989). Evidence for a distinct AZ compartment in the EGL also comes from analysis of *Unc5h3 X* +/+ (Goldowitz et al., 2000) and (small eye) Sey/Sey^Neu X +/+^ chimeras (Swanson and Goldowitz, 2011), and from the effects of several mouse mutations (e.g., *rostral cerebellar malformation*—Eisenman and Brothers, 1998; *NeuroD−/−*—Miyata et al., 1999). Finally, patterns of granular layer and/or EGL gene expression show the AZ/CZ (e.g., acidic FGF, receptor protein tyrosine phosphatase—McAndrew et al., 1998; *Otx-1*—Frantz et al., 1994) and PZ/NZ (e.g., *Otx1*—Frantz et al., 1994; *En2*—Millen et al., 1995; *Tlx3*—Logan et al., 2002; *Lmx1a^+^*—Chizhikov et al., 2010) boundaries. Similarly, neuronal nitric oxide synthase (nNOS: NADPH) is expressed by most granule cells but is entirely absent from those of the NZ (Hawkes and Turner, 1994). While epigenetic interactions with PCs may explain differential gene expression, they cannot account for the spatial distribution of genotypes in the chimeras. We therefore conclude that the cerebellar granular layer has multiple lineage histories and derives from multiple distinct precursor pools either side of lineage boundaries within the RL. It has been established by genetic fate mapping that early born granule cell progenitors (E12.5–E15.5), migrate preferentially into the anterior vermis, whereas later born ones distribute more evenly along the AP axis, and only late-born ones (*circa* E17) populate lobule X (Machold and Fishell, 2005).

**Figure 3:**
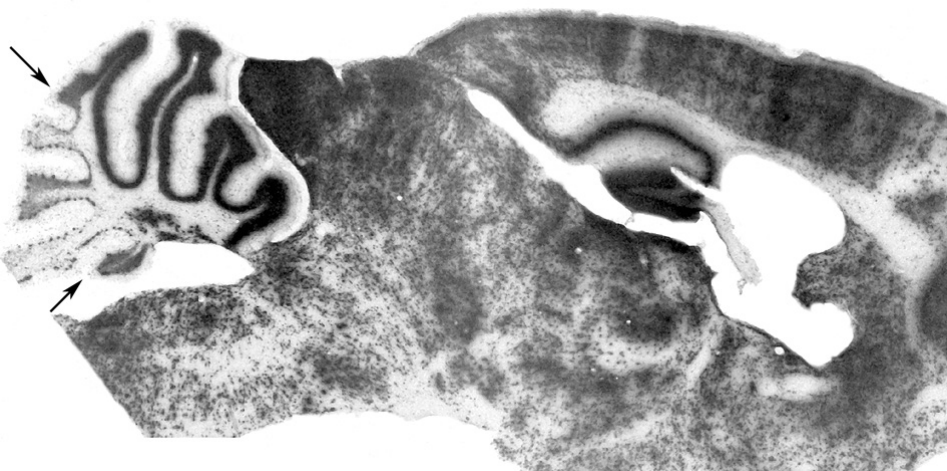
**Compartmentation of the granular layer.** Cell transverse lineage boundaries seen in a β-gal-stained sagittal section through an adult murine embryonic stem cell chimera. The ES-cell-derived granule cells (β-gal+) are concentrated preferentially in the anterior vermis (AZ) with a restriction boundary in lobule VI (AX/CZ: arrow), and in the nodulus with a boundary in the sulcus between lobules IX and X (the PZ/NZ boundary: arrow) [Adapted from Hawkes et al. (1999)].

What determines the location of the granular layer lineage boundaries? While some signals may be intrinsic to early born granule cell progenitors in the RL, the most obvious source of positional information for the developing granular layer (or, more likely, the developing EGL) is the compartmentation of the PCs, and it is therefore noteworthy that the granular layer lineage restriction boundaries roughly align with PC transverse zone boundaries, and suggests that distinct PC compartments may direct the spreading EGL into distinct migratory streams. Consequently, as the EGL comes to cover the cerebellar surface, granule cell lineage discontinuities end up aligned with the PC transverse zone boundaries. Subsequently, both intrinsic differences between granule cell populations and epigenetic interactions between developing granule cells and PCs could contribute to selective patterns of granule cell gene expression. Notably, PCs express extracellular factors during early postnatal development. One of them, Igf-1, is expressed in a pattern very similar to the distribution of zebrin II—PC stripes. Igf-1 acts in an autocrine/paracrine fashion to protect PCs from apoptotic cell death, particularly at birth, and its expression is driven locally by EBF2 expressed by PCs (Croci et al., 2011). Abundant evidence supports a role for Insulin-like growth factor 1 and 2 (IGF1 and IGF2) in central nervous system (CNS) development. IGF1 is predominantly expressed in neurons in a fashion that coincides with outbursts of neural progenitor proliferation, neurite outgrowth, and synaptogenesis (D'Ercole et al., 1996; Bondy and Cheng, 2004; Ozdinler and Macklis, 2006; Fernandez and Torres-Alemàn, 2012). A recent paper indicates that Igf-1, whose levels fluctuate with light-dark cycles, promotes granule cell migration into the GL (Li et al., 2012). Thus, it is conceivable that granule cells in contact with *Igf1*-expressing (zebrin II-) PCs may migrate faster into the GL and thus establish privileged synaptic contacts.

Beyond the restriction of granule cell subtypes to transverse zones, several granule cell markers reveal a much more elaborate parcellation into parasagittal stripes [e.g., in the expression patterns of acetylcholinesterase (Marani and Voogd, 1977; Boegman et al., 1988), cytochrome oxidase (Hess and Voogd, 1986; Leclerc et al., 1990), and nNOS (Yan et al., 1993; Hawkes and Turner, 1994; Schilling et al., 1994)]. It is plausible that this molecular complexity is related to the complex array of somatotopic patches mapped in some cerebellar regions (Welker, 1987; Hallem et al., 1999; Apps and Hawkes, 2009). Finally, perhaps the most curious manifestation of granular layer heterogeneity is the reproducible array of wrinkles in the granular layer (“blebs”) that are seen when ethanol-fixed, paraffin-embedded sections are rehydrated (Hawkes et al., 1997, 1998). The structural basis of blebbing is not known, but it points to the possibility that blebs represent individual cytoarchitectonic units and that the mouse granular layer is subdivided into several thousand modules [reviewed in Hawkes (1997)].

Do these granular layer stripes arise through lineage restriction or are they secondary responses to the local environment (e.g., the type of mossy fiber input or the local PCs)? It is not known but it is difficult to imagine a mechanism by which granule cell stripes form through the targeted migration of granule cell subtypes to hundreds of destinations (although raphes between PC clusters do seem to preferentially guide the descent of immature granule cells to the granular layer: e.g., Karam et al., 2000; Luckner et al., 2001), so it is more plausible that stripe molecular phenotypes among granule cells are secondary responses to local cues. One mechanism might be that granule cells adopt their molecular phenotypes according to the local PC subtype environment through which they migrate (and synapse) during postnatal development (several studies have demonstrated PC influences on granule cell growth and differentiation—e.g., PC-derived sonic hedgehog regulates granule cell proliferation (Wallace, 1999); PC-derived brain-derived neurotrophic factor (BDNF) stimulates granule cell migration—Borghenasi et al., 2002). An alternative mechanism might be that granule cell subtypes are specified by the type of mossy fiber (or UBC) afferent input they receive. As noted earlier (section “Review of cerebellar compartmentation”) mossy fiber terminal fields from different sources and of different molecular phenotypes are restricted to parasagittal stripes that align with PC stripes (e.g., somatostatin-immunoreactive—Armstrong et al., 2009; vesicular glutamate transporter immunoreactive—Gebre et al., 2012). Perhaps differential mossy fiber innervation specifies granule cell subtype. In both cases, PC specification or mossy fiber specification of granule cell subtype, granule cell stripes would naturally also align with PC stripes.

This is consistent with the demonstration by Schilling et al. (1994) that ingrowing mossy fibers may downregulate nitric oxide synthase expression and thereby contribute to the generation of granule cell subtypes.

### Unipolar brush cells

UBCs are glutamatergic interneurons of the granular layer. They receive mossy fiber innervation, in large part from primary vestibular afferents (e.g., Diño et al., 2000, 2001), and project in turn to granule cell dendrites. At least three subtypes of UBC are known, one immunoreactive for calretinin (= CR+ subset), another expressing both the metabotropic glutamate receptor (mGluR1α: Nunzi et al., 2001, 2002) and PLCβ4 (Chung et al., 2009a = the mGluR1α+ subset), and a third expresses PLCβ4 but not mGluR1α (Chung et al., 2009a = the PLCβ4+ subset).

UBCs are born between E15 and P2 (Abbott and Jacobowitz, 1995; Sekerková et al., 2004; Chung et al., 2009b). They appear to have two distinct origins. The majority arise ventrally, possibly in the VZ of the fourth ventricle (e.g., Ilijic et al., 2005) but more likely from the RL since RL ablation in slice cultures significantly reduced the number of UBCs (Englund et al., 2006) and the production of UBCs is decreased in the *Math1* null cerebellum (as mentioned, *Math1* is required for the development of RL derivatives: Machold and Fishell, 2005; Wang et al., 2005). Either way, most UBCs migrate into the developing cerebellar anlage soon after the PCs arrive (E14 onwards: Abbott and Jacobowitz, 1995) and then disperse via the white matter tracts, presumably guided by cues associated with PC axons or afferent projections. In addition, a second, small population of UBCs arises dorsally from the EGL (and presumably the RL) and reaches the granular layer by following the same dorsoventral migratory route as the granule cells (Abbott and Jacobowitz, 1995; Chung et al., 2009b).

Each UBC subset has a characteristic topographical distribution (Braak and Braak, 1993; Floris et al., 1994; Diño et al., 1999; Nunzi et al., 2002; Chung et al., 2008a): all three are concentrated preferentially in the NZ, but mGluRlα+ UBCs are also common throughout the vermis, only occasional CR+ UBCs are seen in the AZ, and the PLCβ4+ subset is very rare in the AZ (Mugnaini and Floris, 1994; Diño et al., 2000; Chung et al., 2009a). Each subset is also loosely restricted to stripes that align with PC stripes (e.g., Chung et al., 2009b: Figure 4). This distribution implies that each UBC subset receives afferent input from a different set of mossy fiber inputs. However, it seems unlikely that UBC subtype phenotypes are secondary to mossy fiber input since when they are allowed to mature as dissociated cells *in vitro*, in the absence of extracerebellar mossy fiber cues, the different phenotypes are all expressed (Anelli and Mugnaini, 2001; Chung et al., 2009a). It is therefore plausible that the restricted distributions of UBC subtypes comes about because each uses different topographical cues to guide their migrations. Experiments using an *Ebf2−/−* mouse support this inference. When *Ebf2* is deleted, many PCs express abnormal molecular phenotypes (a mixture of zebrin II+ and zebrin II−: Croci et al., 2006; Chung et al., 2008b). Notably, anterior vermis PCs adopt features of the posterior vermis. Interestingly, in these mice, the normal restriction of UBCs to the posterior vermis is also lost, and UBC profiles become plentiful in the anterior lobules (Chung et al., 2009b). Because EBF2 is not expressed by UBCs this suggests that the abnormal topography in the mutant is not cell-autonomous but rather secondary to the abnormal PC transdifferentiated phenotype. This is also consistent with the developmental data showing a close association between PCs and UBCs in the perinatal cerebellum (Chung et al., 2009b). Finally, mutations in the Reelin signaling pathway result in a failure of PC cluster dispersal. This results in a corresponding UBC ectopia. For example, in *scrambler* (a *Disabled1* mutant: Sheldon et al., 1997) UBCs are found in association with specific PC ectopic clusters (Chung et al., 2009b). Taken together, the data support the hypothesis that PCs present topographically organized cues to the growth cones of migrating UBCs and thereby restrict their topography.

**Figure 4:**
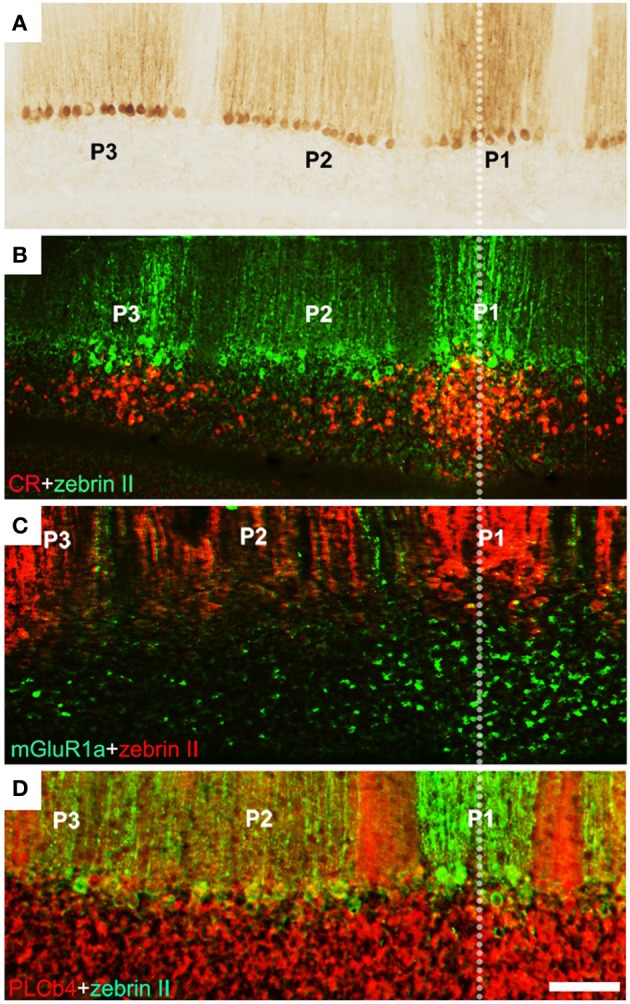
**Unipolar brush cells are restricted at stripe boundaries in the adult mouse cerebellum. (A)** Cerebellar stripe topography is built around PC subtypes. Immunoperoxidase staining of a transverse section through lobule IX for zebrin II reveals three broad stripes of immunoreactive PCs [P1+ at the midline, P2+ and P3+ laterally on either side: for stripe nomenclature, see Sillitoe and Hawkes (2002)]. **(B)** CR+ UBC clusters (red) in lobule IX align with the zebrin II P1+ and P3+ PC stripes (green). **(C)** mGluR1α+ UBCs (green) in lobule IX cluster beneath the midline P1+ PC stripe (red). **(D)** Double immunostaining with anti-PLCβ4 (red) and anti-zebrin II (green) shows that PLCβ 4-immunopositive UBCs are uniformly distributed in cerebellar lobule IX (the combined PLCβ4+ and mGluR1α+ subsets). Scale bars: D = 125 μm **(A–D)** [Adapted from Chung et al. (2009b)].

### Basket and stellate cells

Basket and stellate cells are small inhibitory interneurons of the molecular layer. Whether or not they represent two distinct cell classes or a morphological continuum is unclear (e.g., Schilling et al., 2008): for our purposes we will discuss them together. There is little evidence of distinct subclasses of basket/stellate cells (cyclin D2 expression can distinguish subtypes, but this is amenable to other explanations: Huard et al., 1999). An exception is the study of Chen and Hillman (1993) who showed that a protein kinase Cδ-immunoreactive basket/stellate cell subset in the rat cerebellum is strongly concentrated in the AZ.

All GABA interneuron progenitors transiently activate *Pax2* expression around cell cycle exit. Before homing in on their final location, the young *Pax2*+ interneurons reside for several days in the white matter, progressing in their maturation, and acquiring their final identities. Postmitotic Pax2+ neurons harvested while in the white matter and transplanted heterochronically into a recipient cerebellum invariably give rise to GABA interneurons, but their choice to adopt a CN, granular layer, or molecular layer interneuron fate remains entirely dependent upon the host-specific, extrinsic environment (Leto et al., 2009).

Few inhibitory interneurons are present in the molecular layer at birth. While CN interneurons, are all born between E12 (Florio et al., 2012) and P3, Golgi cells that populate the GL continue to divide until P4 and basket/stellate interneuron progenitors keep proliferating through the second postnatal week [reviewed in Zhang and Goldman (1996); Carletti and Rossi (2008)], according to an inside-out progression (Leto et al., 2006). In rat, the interneurons of the inner molecular layer (= basket cells) are born between P2 and P17 with a peak at P6, while those in the outer molecular layer (= stellate cells) are born between P4 and P19 (peak at P10: Altman, 1972). The morphological evidence of restriction of basket/stellate cell neurites follows a similar continuum. In particular for the cells located deep in the molecular layer (basket) and less so for the more superficial ones (stellate), both the axons and the dendrites tend to be oriented parasagittally. For example, a classic basket cell contacts about 40 PCs. Their terminal fields are ovoid in shape, four times longer than they are wide, and with their long axes aligned parasagittally with the PC stripes (e.g., Eccles et al., 1967; Rakic, 1972; King et al., 1993). This axial ratio is much less for stellate cells. Ever since the work of Szentagothai (1965) it has been recognized that the parasagittal orientation of basket cells represents a substrate for PC lateral inhibition. More recently, and consistent with the morphology, physiological studies both *in vitro* and *in vivo* confirm that the inhibitory fields of basket/stellate cells are confined to a single stripe, with molecular layer inhibition restricted parasagittally (Ekerot and Jörntell, 2001, 2003; Jörntell and Ekerot, 2002; Gao et al., 2006; Dizon and Khodakhah, 2011; etc.: the functional implications of reciprocal inhibition between interneurons within a microzone have recently been reviewed—Jörntell et al., 2010).

**Figure 5:**
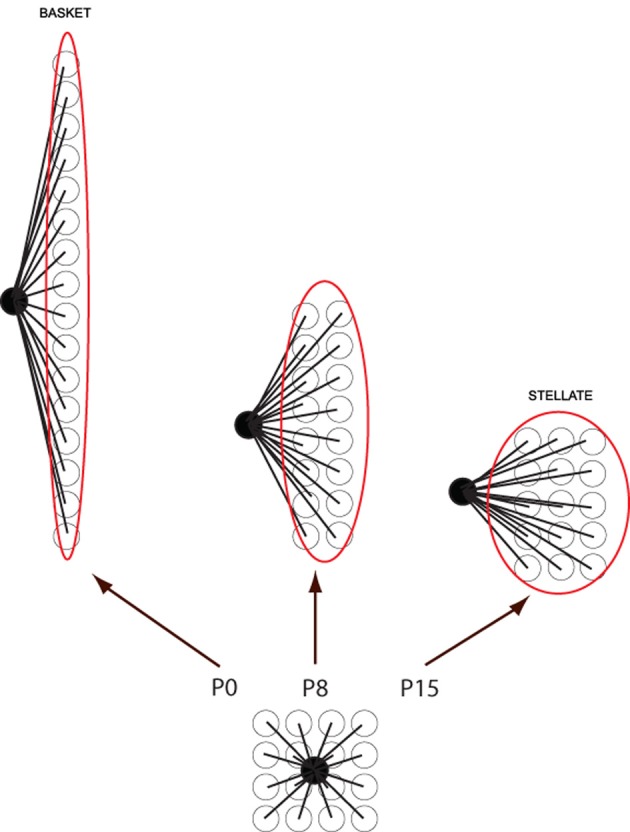
**Cartoon to show how basket/stellate cell parasagittal orientations might arise.** Newly migrated basket/stellate cells enter the molecular layer and synapse on a small group of PCs. As the PCs extend parasagittally to form adult stripes, the basket/stellate cell arbors extend with them. As a result the shape of the final axonal arbor depends on the time they arrive: the earlier they enter the molecular layer, the more extended the arbor (= basket cell), the later they enter, the less it is extended (= stellate cells).

How do basket/stellate cells acquire their parasagittal orientations? First, they are born too late to interact with embryonic PC clusters (from P2 to P19: and anyway there is little evidence of subtype specification). However, the parasagittal orientation of basket cell axonal arbors can still be explained by PC rostrocaudal spreading. Molecular layer interneurons invade the immature molecular layer randomly from the white matter. Once in the molecular layer they contact a local cluster of some 40 PCs. In the course of the next 3 weeks, these PCs gradually disperse rostrocaudally, so that the cerebellar cortex extends more than 10-fold in rostrocaudal extent with almost no change in width. As a result, the basket/stellate cell terminal field becomes a short, parasagittal PC stripe (Figure 5). It is not clear to what extent the basket/stellate terminal fields are restricted to particular stripes. It could be that there are as yet unrecognized subtypes (as for UBCs, for example), or that secondary pruning refines their arbors, or the restriction could be purely statistical. In any case, PC dispersal would result in a continuum of terminal field shapes: the earliest-born interneurons enter the molecular layer first and therefore develop the most extended parasagittal terminal fields (basket cell); the later-born interneurons have progressively more symmetrical terminal fields (e.g., stellate cells—Sultan and Bower, 1998).

### Golgi cells

Golgi cells are large interneurons of the granular layer (Palay and Chan-Palay, 1974). Golgi cell apical dendrites ramify through the molecular layer and are contacted primarily by the axons of granule cells (e.g., Geurts et al., 2003). Their axonal terminals contact granule cell-mossy fiber glomeruli. Five distinct classes of Golgi cell have been identified based on morphology and differential expression patterns (various combinations of glycinergic, gabaergic, mGluR2+/−, and neurogranin+/−: Simat et al., 2007).

The origin of Golgi cells is controversial. On the one hand, Popoff (1896) and Athias (1897) suggested the EGL was the origin of these large neurons. This interpretation was supported by the more recent studies of Hausmann et al. (1985), who used cerebellar transplantation to provide experimental evidence that Golgi cells originate from the EGL. More recently, Chung et al. (2011) also identified a small, unique population of ZAC1-immunopositive Golgi cells, restricted to the posterior zone of the cerebellum that appears to derive from the EGL between E13 and E16. On the other hand, Ramon y Cajal (1911) and Altman and Bayer (1977) both concluded that Golgi cells derive from the ventricular neuroepithelium. Zhang and Goldman (1996) reached the same conclusion based on retroviral lineage tracing data. By this view Golgi cells, as all other GABA interneurons of the cerebellum, originate from *Ascl1*+ progenitors that delaminate from the VZ into the prospective white matter, exit the cell cycle, and activate *Pax2*. It seems probable that both explanations are correct, and that two distinct populations of Golgi cells are present in the adult cerebellum.

With the exception of the restriction to the PZ of the ZAC1+ population (Chung et al., 2011) nothing is known of the localization of Golgi cell subtypes to particular transverse zones or lobules. However, a different sort of patterning does occur: the apical dendrites of Golgi cells show restriction at parasagittal PC stripe boundaries (Sillitoe et al., 2008: Figure 6). Golgi cell apical dendrites contact the parallel fiber axons of granule cells in the molecular layer. By using different markers of Golgi cell dendrites and a selection of stripe antigens, the apical dendritic arbors of Golgi cells were studied in the vicinity of PC stripe boundaries. The conclusion was clear—fewer than 3% of Golgi cell dendrites cross a PC stripe boundary (Sillitoe et al., 2008). The mechanisms that restrict Golgi cell dendritic arbors are speculative. They might be prevented from crossing stripe boundaries by structural barriers (such as those reported in the somatosensory cortex—Faissner and Steindler, 1995). However, no such barriers to neurite extension are known and other axons (e.g., parallel fibers) cross parasagittal stripes unhindered. Alternatively, Golgi cell dendritic arbors may be restricted, via adhesion molecules or attractive/repulsive extracellular cues, as they develop in concert with the PC dendrites (e.g., Hekmat et al., 1989; Nagata and Nakatsuji, 1991). In this model, the newly born Golgi cells migrate via the white matter into the embryonic PC clusters (Zhang and Goldman, 1996) where they contact the nascent PC dendrites. Subsequently, as the PC clusters disperse into adult stripes, individual Golgi cell dendritic arbors would automatically become restricted to one side of a boundary Subsequently, as the granule cells mature, the Golgi cell dendrites would displace from the PCs and synapse with local parallel fibers. In this way they would retain their original topographical restriction.

**Figure 6:**
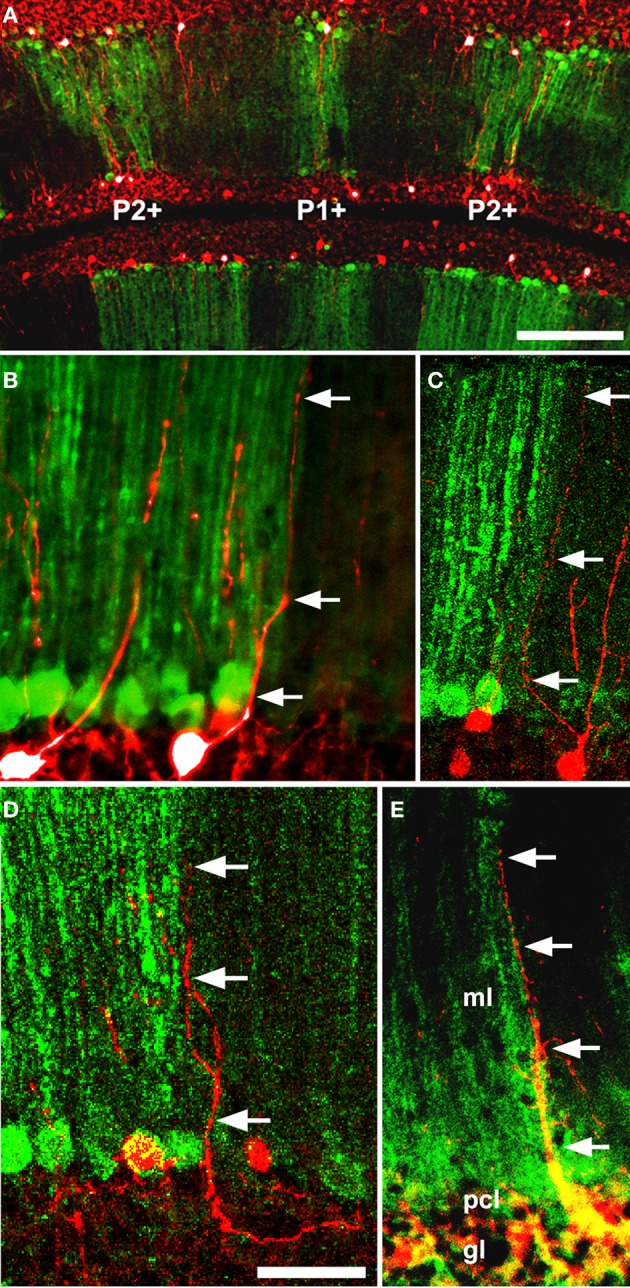
**Five examples (A–E) of double immunofluorescence for a GlyT2-EGFP transgene (Golgi cell dendrites: Zeilhofer et al., 2005) and anti-PLCβ4 (PC stripes: Sarna et al., 2006) in the adult mouse cerebellum reveals that Golgi cell dendrites are restricted at Purkinje cell stripe boundaries.** Two examples of GlyT2-EGFP+ Golgi cell dendrites (green: arrowheads) in the vicinity of a PLCβ4+/− stripe boundary (red): in 68 cases examined, the dendrite never crossed between stripes. Abbreviations: ml, molecular layer; pcl, Purkinje cell layer; gl, granular layer. Scale bar = 50 μm [From Sillitoe et al. (2008)].

## Purkinje cell architecture generates interneuron restriction

We have reviewed the evidence that cerebellar interneurons show anatomical and molecular restriction to zones and stripes. The general hypothesis presented is that these restrictions come about through interactions with the PC architecture. In this light, it is worthwhile to recall briefly the hypothesis to explain how climbing and mossy fiber afferents become aligned with PC stripes. First, the afferent fiber growth cones make direct contacts with specific embryonic PC clusters (e.g., Sotelo and Wassef, 1991; Grishkat and Eisenman, 1995; Chédotal et al., 1997; Sotelo and Chédotal, 2005). Subsequently, the clusters disperse into stripes triggered by Reelin signaling. As a result, the PC layer extends in the rostrocaudal plane, the clusters transform into stripes and, because the afferent terminal fields are carried along, they too form stripes, which are aligned with specific PC stripes. In the case of mossy fibers, as the granular layer matures, the mossy fibers detach from their embryonic PC targets (Mason et al., 1990) and synapse instead on local granule cell dendrites. Hence, although the mossy fibers no longer directly contact PCs their terminal fields remain aligned with specific PC parasagittal stripes.

This hypothesis is straightforwardly adaptable to the interneurons of the cerebellar cortex. First, the developing EGL spreads over the surface of the cerebellar anlage, restricted by cues from the underlying PCs (section “Zone and stripe boundaries restrict cerebellar interneurons”). As a result, different EGL lineages become aligned with boundaries between different PC transverse zones (Ozol and Hawkes, 1997; Hawkes et al., 1999, etc.). The lineage boundaries are also expression boundaries, but it is not clear whether these are also lineage restricted or if they are secondary responses to local PC cues. Developing Golgi cells and most UBCs access the PC clusters via the white matter tracts (Leto et al., 2006, 2009) and associate with specific PC clusters (e.g., Chung et al., 2009b). Therefore, as the PC clusters disperse into stripes the Golgi cells and UBCs move with them, just as for mossy fiber afferent terminal fields, and therefore also become restricted to specific stripes and zones. Subsequently, they mimic the mossy fibers and relocate from the PCs to the granular layer as it matures. This also explains how Golgi cell apical dendrites become restricted to particular PC stripes (Sillitoe et al., 2008). The dispersal of embryonic PC clusters into stripes continues roughly during the first three postnatal weeks (in mice). Although basket/stellate cells are born too late to interact with the embryonic PC clusters they benefit from cluster dispersal to orient their axon arbors parasagittally. Once *in situ*, interneurons differentiate in response to local environmental cues (e.g., granule cell stripes of nNOS—Hawkes and Turner, 1994).

Finally, it is interesting to speculate why parallel fibers appear to be the sole exception: why are parallel fibers not restricted? One possibility is that it is important that they are not. Parallel fibers are several millimeters long (e.g., Brand et al., 1976) and as they run orthogonal to the PC stripes, they necessarily intersect many stripes, of many different subtypes. In a nutshell, if PC boundaries were to restrict parallel fibers there would be no parallel fibers! A subtler question is whether they synapse with all the PC dendritic arbors that they pass through. This issue has not been studied experimentally. One scenario is that parallel fibers have a primary function to distribute MF afferent input widely across multiple neighboring stripes, in which case they might be expected to synapse promiscuously with *all* stripes they encounter, at least initially. How that input is used is another matter. One possibility is that PCs synapse with every PC they intersect but only a subset of those synapses is active: in one study, a large fraction of parallel fiber-PC synapses were found to be silent (Brunel et al., 2004), so sculpting in this fashion is entirely plausible.

### Conflict of interest statement

The authors declare that the research was conducted in the absence of any commercial or financial relationships that could be construed as a potential conflict of interest.
